# Advances in Dystrophinopathy Diagnosis and Therapy

**DOI:** 10.3390/biom13091319

**Published:** 2023-08-28

**Authors:** Fawzy A. Saad, Gabriele Siciliano, Corrado Angelini

**Affiliations:** 1 Department of Gene Therapy, Saad Pharmaceuticals, Juhkentali 8, 10132 Tallinn, Estonia; 2 Department of Clinical and Experimental Medicine, Pisa University School of Medicine, Via Paradisa 2, 56100 Pisa, Italy; gabriele.siciliano@unipi.it; 3 Department of Neurosciences, Padova University School of Medicine, Via Giustiniani 5, 35128 Padova, Italy; corrado.angelini@unipd.it

**Keywords:** dystrophinopathies, Duchenne muscular dystrophy, Becker muscular dystrophy, dilated cardiomyopathy, serum creatine kinase, pharmacological therapy, gene drugs, gene therapy, CRISPR gene editing, prime gene editing

## Abstract

Dystrophinopathies are x-linked muscular disorders which emerge from mutations in the *Dystrophin* gene, including Duchenne and Becker muscular dystrophy, and dilated cardiomyopathy. However, Duchenne muscular dystrophy interconnects with bone loss and osteoporosis, which are exacerbated by glucocorticoids therapy. Procedures for diagnosing dystrophinopathies include creatine kinase assay, haplotype analysis, Southern blot analysis, immunological analysis, multiplex PCR, multiplex ligation-dependent probe amplification, Sanger DNA sequencing, and next generation DNA sequencing. Pharmacological therapy for dystrophinopathies comprises glucocorticoids (prednisone, prednisolone, and deflazacort), vamorolone, and ataluren. However, angiotensin-converting enzyme (ACE) inhibitors, angiotensin receptor blockers (ARBs), and β-blockers are the first-line to prevent dilated cardiomyopathy in dystrophinopathy patients. Duchenne muscular dystrophy gene therapy strategies involve gene transfer, exon skipping, exon reframing, and CRISPR gene editing. Eteplirsen, an antisense-oligonucleotide drug for skipping exon 51 from the *Dystrophin* gene, is available on the market, which may help up to 14% of Duchenne muscular dystrophy patients. There are various FDA-approved exon skipping drugs including ExonDys-51 for exon 51, VyonDys-53 and Viltolarsen for exon 53 and AmonDys-45 for exon 45 skipping. Other antisense oligonucleotide drugs in the pipeline include casimersen for exon 45, suvodirsen for exon 51, and golodirsen for exon 53 skipping. Advances in the diagnosis and therapy of dystrophinopathies offer new perspectives for their early discovery and care.

## 1. Introduction

**Figure 1:**
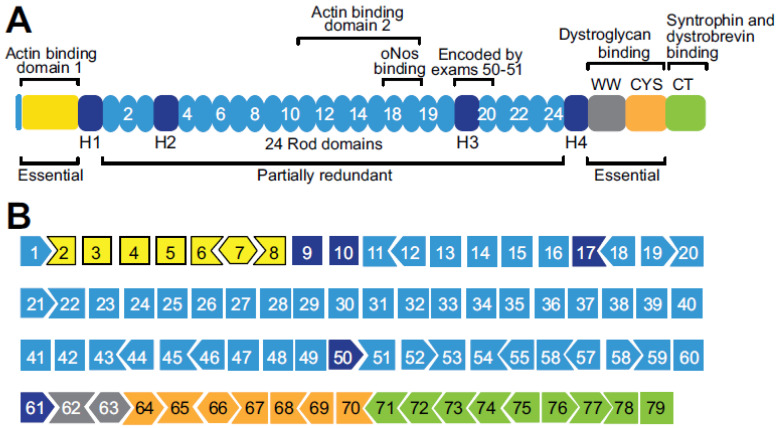
*Dystrophin* gene exon structure and protein functional domains. (**A**) Schematic of dystrophin protein showing functional domains. Hinge (H), WW (Rsp5 domain), cysteine-rich (CYS), and carboxyl-terminal (CT) domains. Rod domain spectrin repeats are numbered 2 through 24. (**B**) Exons splicing patterns of the human dystrophin gene. Colors correspond to functional domains of the protein in (**A**). Shapes of exons indicate whether splicing between adjacent exons maintains the contiguous ORF of the protein when the shapes fit together like pieces of a puzzle. Reproduced with permission from Eric N. Olson [5].

Molecular cloning of the *Dystrophin* gene in 1985 [1,2] has revealed that its 14 kilobase messenger RNA comprises 79 exons extending over 2.4 megabases (2.4 centimorgans) of the human chromosome X [3,4]. The dystrophin protein contains four domains [5]: the amino-terminal, the rod, the cysteine-rich, and the carboxy-terminal domains (Figure 1A).

Dystrophin and utrophin are muscle cytoskeletal proteins with similar molecular masses of 420 and 395 kDa, respectively. Furthermore, utrophin shares 80% homology with the dystrophin carboxy-terminal domain [6,7].

Approximately 75% of *Dystrophin* gene mutations are intragenic deletions (65%) or duplications (10%), while the remaining 25% are nucleotide variants, including nonsense and missense mutations, small insertions/deletions (indels), or splicing alterations [8,9]. Out of frame exon deletions/duplications and nonsense mutations generate transcripts with premature stop codons which would be degraded through a nonsense-mediated mRNA decay pathway [10,11]. Figure 1B shows exons splicing patterns of the *Dystrophin* gene.

The absence or deficiency of dystrophin due to mutations in the *Dystrophin* gene leads to spectrum of dystrophinopathies including Duchenne muscular dystrophy (DMD), Becker muscular dystrophy (BMD), and dilated cardiomyopathy (DCM) in humans and animal models [12,13]. Cardiomyopathy is a common feature of DMD patients and influences the prognosis of the disease [14]. The prevalence of dilated cardiomyopathy in DMD patients rises from 59% to 90% depending on the age of the patients [15], and may reach 61% in BMD patients [16]. However, carriers of *Dystrophin* gene mutations may represent a rare distinct form of dilated cardiomyopathy without skeletal muscle abnormalities [17].

Dystrophinopathies are associated with elevated levels of serum creatine kinase (CK) beyond the background of metabolic myopathies [18,19,20]. DMD is the most severe phenotype, which usually manifests in childhood with a sequence of muscle degeneration leading to a loss of mobility before the teenage years. Conversely, BMD is a late-onset entity, therefore, patients with a mild BMD spectrum display symptoms after the age of 30 and stay mobile even into their 60s. However, left ventricular dilation and congestive heart failure are common causes of morbidity and a prevalent cause of death for BMD patients, which may occur early in their 40s. Melacini and colleagues illustrated that the deletion of exon 49 from the *Dystrophin* gene is associated with cardiac manifestation, which is characterized in BMD patients by early right ventricular involvement related or not to left ventricular weakening [21].

Dystrophinopathies are inherited in a sex-linked recessive manner, mainly affecting boys due to their single copy of chromosome X. As carrier girls have two copies of chromosome X, they are at increased risk for dilated cardiomyopathy. Although DMD and BMD are the common types of dystrophinopathies in boys, isolated dilated cardiomyopathy, myalgia, cramps, rhabdomyolysis, hyperCKemia (elevated serum creatine kinase), are less common manifestations in carrier girls [22]. However, about 8–18% of carrier girls present with dilated cardiomyopathy, which could vary to certain degree depending on whether the carrier girl manifests a DMD or BMD phenotype [20,23,24,25,26,27]. This review highlights the advances in dystrophinopathy diagnosis and therapy.

## 2. Diagnosis Technology

Prior to the molecular diagnosis era, diagnosis of muscular dystrophy patients and their carrier mothers has mainly relied on elevations of serum creatine kinase [28], and to lesser degree on muscle histopathology [29] and haplotype (pedigree) analysis of the *Dystrophin* gene using restriction fragment length polymorphisms [30] or short tandem repeat polymorphisms [31].

### 2.1. Serum Creatine Kinase Assay

More than six decades ago, investigative studies provided evidence that serum creatine kinase (CK) is superior to other enzymes like aldolase, lactic dehydrogenase, glutamic oxalacetic transaminase, glutamic pyruvic transaminase, and phosphohexoseisomerase for the biochemical diagnosis of muscular dystrophy patients and their carrier mothers [28,32,33,34,35], allowing for the detection of preclinical cases of muscular dystrophy and disease prediction in infancy. Since then, elevation of serum creatine kinase (CK) was the prominent preclinical diagnostic tool and is still in use in the molecular diagnosis era [36].

Duchenne muscular dystrophy is always coupled with high levels of serum creatine kinase [20]. Yasmineh and colleagues have reported that serum creatine kinase in DMD patients reached around 867 U/L compared to 28 U/L of the healthy control group, which is thirty-one-fold higher than healthy subjects [37]. However, serum creatine kinase assay has the potential to diagnose up to 71% of DMD carriers [33]. In fact, normal serum creatine kinase levels in two DMD carriers with muscle histological abnormalities have been reported [38].

The molecular diagnosis era started with the cloning of the *Dystrophin* gene in 1985 [1,2]. Since then, the diagnosis of muscular dystrophy patients and their carrier mothers has relied on haplotype analysis, Southern blot analysis, immunological analysis, multiplex polymerase chain reaction (PCR), multiplex ligation-dependent probe amplification, Sanger DNA sequencing, and next generation DNA sequencing.

### 2.2. Haplotype and Southern Blot Analyses

Molecular diagnosis of DMD was initiated four decades ago with haplotype (pedigree) analysis, using restriction fragment length polymorphisms (RFLP) related to the *Dystrophin* gene [39,40]. Furthermore, the use of Southern blotting and complementary DNA (cDNA) probe hybridization has detected several intragenic deletions and duplications in the *Dystrophin* gene [41,42,43,44,45,46,47,48,49].

### 2.3. Diagnostic Methods on Muscle Tissue

Immunohistochemical analysis of muscle biopsy cryosections reveals that human dystrophin antibodies react with an antigen in skeletal muscle sarcolemma. This immunoreactivity is faint or absent in muscle fibers from DMD patients compared to the muscle fibers of healthy subjects. On immunoblots, dystrophin antibodies react with 400 kDa protein extracts of normal human muscle [50,51,52]. Protein truncation tests revealed that about 73% (19 out of 26) of BMD patients show a truncated dystrophin of abnormal molecular weight [52,53,54], leading to the presence of normal and shorter dystrophins in the muscle fibers of BMD asymptomatic carriers. However, in case of duplication, BMD patients show longer and normal dystrophins [44]. Also, a screening of 62 Becker muscular dystrophy patients revealed that 35 of them had dystrophin abnormalities [55]. Currently, there are six anti-human dystrophin antibodies for Western blotting that recognize the different domains of dystrophin; one is polyclonal and five are rabbit monoclonal antibodies available from various commercial sources. RNA sequencing (RNA-seq) is a valuable approach for dystrophin mutation detection [56,57,58,59].

### 2.4. Multiplex Ligation-Dependent Probe Amplification

Multiplex ligation-dependent probe amplification (MLPA) is widely employed to examine exonic duplications/deletions (dupdels) within the 79 exons of the *Dystrophin* gene [60,61,62,63,64,65,66,67], detecting up to 70% of exonic alterations. However, genetic diagnosis of the remaining 30% of DMD/BMD patients requires point mutations screening and DNA sequencing. Sanger DNA sequencing of the entire *Dystrophin* gene obtained from the analysis of reverse transcription (RT-PCR) from muscle *Dystrophin* mRNA (cDNA) is a powerful approach for detecting nucleotide alterations within the transcript of the *Dystrophin* gene [56,57].

### 2.5. Multiplex PCR

For more than a decade, the standard clinical diagnosis relied on the conventional multiplex PCR for a selected number of *Dystrophin* exons within the proximal (exons 3–9), and the distal (exons 45–55) deletion hotspots [68]; nonetheless, this multiplex PCR platform holds the power to confirm the presence of exonic deletions in about 98% of DMD/BMD boys [69,70]. Although effective and economic, this multiplex PCR platform was imperfect; because it did not include all the 79 exons of the *Dystrophin* gene, leaving the deletions border undefined and the reading frame ambiguous in many patients [71].

Soon after, semiquantitative multiplex PCR was able to detect intragenic duplications in DMD patients and girls carrying intragenic deletions within the *Dystrophin* gene [72,73]. About two decades later, semiquantitative fluorescent multiplex PCR for deletions and duplications detection was achieved [57]. The high-density single strand PCR-based comparative genomic hybridization (CGH) array represents an effective high-throughput tool (DMD-CGH array) to detect *Dystrophin* gene exon deletions/duplications [74,75].

### 2.6. Point Mutations Screening

If the results of multiplex PCR and MLPA analyses do not reveal intragenic alterations (single or multiple exon deletions or duplications—dupdels), the next step is to screen PCR products for nucleotide alterations (point mutations) including nucleotide substitutions, deletions, or insertions using denaturing gradient gel electrophoresis [76,77], single strand confirmation polymorphism analysis [78,79], double strand confirmation analysis [80,81], or DNA fingerprinting [82,83,84].

If the point mutations screening shows electrophoretic mobility alterations of certain PCR products (amplicons), the next step is to sequence these amplicons to identify nucleotide alterations. Furthermore, rapid direct sequencing of *Dystrophin* gene exons and flanking intronic regions, which is necessary to detect mutations affecting splice sites, became available in 2003 [16,85].

### 2.7. Next Generation DNA Sequencing

The next generation DNA sequencing (NGS) platform is a valuable tool for the molecular diagnosis of dystrophinopathies [86,87,88,89,90,91]. The NGS platform combines a DNA sequencing apparatus (NovaSeq 6000, sequencer) and results analysis gear (SeqNext software, version number is 3.5.0). The NGS platform is able to simultaneously detect intragenic and nucleotide alterations of libraries obtained with the DMD MASTR kit (Agilent Technologies, Cheadle, UK).

The combination of MLPA and NGS is a valuable approach for detecting mutations in the *Dystrophin* gene in Peruvian patients suspected of muscular dystrophies [92]. Moreover, a comprehensive NGS assay for sequencing the entire 2.2 Mb *Dystrophin* gene holds the power to detect all copy number and sequence variants in both patients and carrier females [93].

## 3. Pharmacological Therapy

Since the first description of Duchenne muscular dystrophy in 1867 [94], various pharmacological efforts have failed to alter the natural course of the disease [95,96]. Recent updates of the pharmacological therapy for Duchenne muscular dystrophy are reported elsewhere [97].

### 3.1. Skeletal Muscle Care

The mainstay therapy of DMD patients is glucocorticoids (prednisone, prednisolone, and deflazacort), which target the glucocorticoid receptor to exert anti-inflammation effects by suppressing the NF-κB signaling pathway [98,99,100]. Glucocorticoids are usually administered as daily or intermittent doses; however, glucocorticoids have different efficacy and remarkable side effects [99,101,102], including weight gain, osteoporosis, cataracts, hypertension, and stunted bone growth [103,104,105,106,107]. Bonifati and colleagues suggest that the 1220 A to G (Asn363Ser—N363S) polymorphism in the *Glucocorticoid receptor* (*GR*) gene has a definite modulating effect on steroid response in DMD patients by inducing a long-term sensitivity to glucocorticoids [108].

In a randomized double-blind controlled trial, 28 DMD patients were treated with either deflazacort 2.0 mg/kg or placebo on alternate days. After 6 months of therapy, the deflazacort group showed significant progress in climbing stairs, rising from a chair, Gower’s maneuver, and walking. Moreover, these motor outcomes continued to meliorate during a two-year follow-up period. Additionally, the loss of ambulation of the deflazacort group was delayed for 12.7 months compared to placebo—33.2 versus 20.5 months, respectively [109]. Wissing and colleagues suggest that the cyclophilin inhibitor (Debio-025) is more effective than prednisone in reducing skeletal muscle pathology in the DMD mouse model [110], which is due to its ability to desensitize mitochondrial permeability pore and successive cellular necrosis. This observation suggests a latent mitochondrial dysfunction in DMD myoblasts [111].

Currently vamorolone (VBP15), an innovative steroid, is being investigated as a potential alternative to corticosteroids (glucocorticoids and mineralocorticoids), aiming at maintaining the corticosteroids’ efficacy profile while diminishing their side effects [112,113,114]. Ataluren is approved in several countries for DMD therapy. Ataluren (Translarna—PTC124) is a disease-modifying molecule for stop codon read-through therapy, which could help up to 10–15% of the DMD patients carrying nonsense mutations plus those carrying out of frame mutations [20,115,116,117,118]. However, there is no pharmacological drug that can compensate for the lack of dystrophin in muscle fibers [96,97].

Corticosteroids (prednisone, prednisolone, and deflazacort) stabilize muscle strength for some time [106]. Although corticosteroid therapy improves muscle strength and function for DMD boys aged 5 to 15 years, their therapeutic efficacy in BMD is less obvious. Moreover, intermittent glucocorticoids combined with continuous oral steroid therapy significantly improve myocardial function in DMD, but not in BMD patients [119]. Merlini and colleagues reported that early corticosteroid treatment increases quadriceps muscle strength and prolongs the mobility of DMD boys [120]. Barp and colleagues identified a putative predictive value of the LTBP4 rs10880 genotype for delaying the onset of dilated cardiomyopathy with steroid therapy, which could help in deciding whether and how long to preserve therapy in non-ambulatory patients [121].

### 3.2. Cardiologic Care

Angiotensin-converting enzyme (ACE) inhibitors, angiotensin receptor blockers (ARBs), and β-blockers are the first-line cardioprotective prescriptions to prevent DMD cardiac manifestations [121,122,123,124]. While ACE inhibitors are used with or without beta blockers for cardiomyopathy in muscular dystrophy patients, congestive heart failure is treated with diuretics and oxygen. Nevertheless, cardiac transplantations are usually offered to DMD patients and symptomatic carriers with severe dilated cardiomyopathy [13,125,126]. Angiotensin II is involved in the fibrotic process of skeletal muscle and heart [127]. The ACE inhibitor perindopril is associated with a lower mortality in young DMD patients with cardiomyopathy [128].

## 4. Standard Multidisciplinary Care

Duchenne muscular dystrophy affects multiple organs, thus, besides physiotherapy, a multidisciplinary approach for pulmonary, cardiac, and orthopedic care will be adopted. Duchenne muscular dystrophy patients suffer from skeletal muscle degeneration as well as lung and heart function limitations. However, advances in pulmonary care have significantly reduced respiratory complications [129]. The combination of yoga and early age physiotherapy intervention improves pulmonary function in children with DMD [130]. Also, home exercise plays an important role in preventing early complication in patients with muscular dystrophy [131] and may increase their bone mass [132]. There is some general dietary advice such as on the consumption of micronutrients (multivitamins, calcium, vitamin D, high protein diet with low fat and carbohydrates) which should be part of a good dietary practice. However, DMD patients should always consult with their physicians for their nutrient requirements [129].

## 5. Gene Therapy

Duchenne muscular dystrophy gene therapy strategies are comprehensively reported elsewhere [95,97]. Eteplirsen, an antisense-oligonucleotide drug for exon 51 skipping from the *Dystrophin* gene, is available on the market after FDA approval in 2017 [133], however, there are reservations about its efficacy. Other FDA-approved exon skipping drugs include ExonDys-51 for exon 51, VyonDys-53 and Viltolarsen for exon 53 and AmonDys-45 for exon 45 skipping [134]. Exon 51 skipping offers gene therapy for up to 14% of DMD patients [133]. Other antisense oligonucleotide drugs in the pipeline include casimersen for exon 45, golodirsen for exon 53, and suvodirsen for exon 51 skipping [135].

Prime gene editing alone is able to correct up to 89% of the genetic mutations causing genetic diseases [136]. Dystrophin restoration therapies have been developed using synthetic antisense oligonucleotides drugs (genetic medicine, genetic drugs, or gene drugs) to restore the reading frame by exon skipping or exon reframing for individuals with specific pathogenic variants in the *Dystrophin* gene [137,138,139]. Bello and colleagues conclude that patients with deletions ending at exon 51 (del X-51) or an exon 48 isolated deletion (del 48) have mild or asymptomatic BMD, while deletions starting at exon 45 (del 45-X) cause relatively severe weakness [16]. Similar to deletion of exons 45–55 [140], deletion of exons 10–25, 10–29, and 11–30 show dystrophin quantities similar to control [16], providing models for exon skipping of deletions within these exonic intervals.

## 6. Discussion

**Figure 2:**
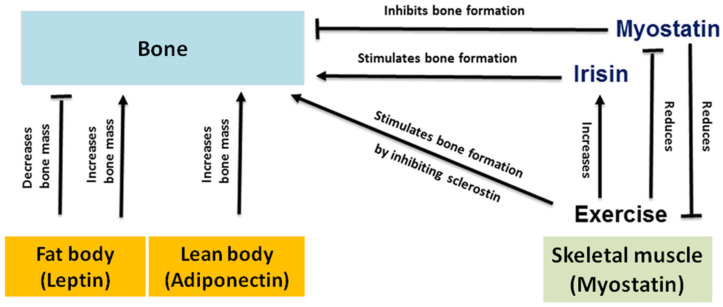
The integrative physiology of muscle, bone, and adipose. Myostatin is a negative regulator of skeletal muscle and bone mass. Myostatin exerts its effects on bone mass by inhibiting bone formation through stimulating the bone resorption pathway and on muscle through the inhibition of myogenesis by downregulating the master transcription factor MyoD. However, exercise leads not only to reduction of myostatin and SOST expression, but also simultaneously increases the expression of irisin, which stimulates the bone formation pathway. While adiponectin increases bone mass, leptin reduces bone mass. The dual effect of leptin signaling on bone differs considerably between axial and appendicular regions. Image is modified from Saad 2020 [132] with License Number 5500950265743 from John Wiley and Sons.

The association between muscular and bone dystrophy was reported seven decades ago [141,142]. Figure 2 portrays the integrative physiology of muscle, bone, and adipose. DMD is associated with low bone mineral density and bone fractures—bone dystrophy [143], which is exacerbated by the use of corticosteroids. In contrast, DMD patients treated with vamorolone show normal bone growth and increase in or normal serum osteocalcin; a bone formation biomarker [113,144]. So far, the use of bisphosphonates to prevent bone loss in DMD patients has not been investigated in clinical trials [102].

Unlike Southern blotting, PCR cannot resolve long stretches of DNA duplications or deletions like the large restriction fragments of multi-kilobase produced by certain restriction enzymes [49]. Haplotype analysis could not predict disease or carrier status with certainty because of the chance for meiotic recombination [39] within a large gene like *Dystrophin* (2.4 centimorgans). Likewise, short tandem repeat polymorphisms may be an inaccurate diagnostic tool due to recombination within the large gene [31]. Serum CK is age-dependent and has a high false-negative rate [145]. Therefore, serum CK is not the ideal test for carrier diagnosis since it may show normal results [20]. However, the relatively delayed onset of the disease in females or males without serum CK elevations suggests specific molecular events [146]. Also, the serum CK assay is simple, inexpensive, and very sensitive once DMD symptoms emerge [147].

*Dystrophin* cDNA analysis by RT-PCR would be required when a splicing mutation is identified within the *Dystrophin* transcript, which may abolish a real splicing site or create a new splicing site. Such a splice mutation may lead to skipping a real exon or creating a cryptic exon [57]. Similar to MLPA, RNA studies are required in order to validate the significance of high-density single strand PCR-based CGH array (DMD-CGH array) findings [75], which is due to their inability to investigate uncoding regions. As predicted, the DMD-CGH array has failed to detect small intronic splicing mutations, for which mRNA analysis was required.

Early multiplex PCR of 19 exons detects about 95% of exonic deletions; however, one of the pitfalls of this multiplex PCR is its inability to detect deletions in carrier females, who have one intact copy of the gene. This pitfall was overcome with the advent of semiquantitative multiplex PCR [72,73]. The development of semiquantitative fluorescent multiplex PCR and the use of the CGH gene-specific array have allowed the detection of dupdels in carrier girls [148]. However, immunoblot analysis can identify abnormalities in dystrophin in the absence of detectable PCR deletions within the gene [149]. Although multiplex ligation-dependent probe amplification holds the power to detect 70% of *Dystrophin* gene intragenic dupdels [60,61,62,63], it is unable to detect nucleotide alterations (point mutations). Therefore, point mutations screening and Sanger DNA sequencing are necessary for detecting the missing 30% of nucleotide alterations. Several approaches are available for point mutations screening; nonetheless, a common disadvantage to these approaches are their sensitivity limitations [85]. Rapid direct DNA sequencing holds the power to detect *Dystrophin* gene mutations in 70% of patients who did not have exonic alterations [85]. However, immunoblot analysis is a rapid and cost-effective alternative for detecting dystrophin alterations in dystrophinopathies [150,151,152,153]. The development of next-generation sequencing technology has revolutionized the diagnosis of neuromuscular disorders [154]. Advances in molecular diagnosis technologies offer unparalleled opportunity to precisely diagnose DMD patients.

While there are no pharmacological drugs that can compensate for the absence of dystrophin in muscle, gene therapy is promising to correct *Dystrophin* gene alterations at their DNA root. Pharmacological drugs including corticosteroids and vamorolone are palliatives which treat the symptoms rather the cause of the disease rooted in the genomic DNA; nonetheless, palliative therapies have extended the average lifespan of patients, from 20 plus years to about 40 years [155].

Ataluren is a disease-modifying molecule for stop codon read-through therapy; thus it restores dystrophin in the muscles. Gene transfer, exon reframing, exon skipping, and CRISPR gene editing hold the promise to correct *Dystrophin* gene mutations at their roots. Currently, Eteplirsen, a gene drug, is available on the market for exon 51 skipping. Other gene drugs are FDA-approved including ExonDys-51 for exon 51, VyonDys-53 and Viltolarsen for exon 53, and AmonDys-45 for exon 45 skipping [134]. Exon 51 skipping offers gene therapy for up to 14% of DMD patients [133]. Furthermore, several antisense oligonucleotide drugs are in the pipeline including casimersen for exon 45, golodirsen for exon 53, and suvodirsen for exon 51 skipping. The lack of muscle-specific delivery, the development of AAV neutralizing antibodies, and the poor control of transgene expression are major obstacles for the translation of gene therapy to patients. However, the use of autologous exosomes to deliver CRISPR gene editing cargo or camouflaging the viral capsids to evade immune detection, and the use of muscle-specific promoter to drive the transgene expression shall alleviate these obstacles [97].

## 7. Conclusions

More than one and a half centuries have elapsed since the first description of Duchenne muscular dystrophy; nonetheless, currently there is no pharmacological cure for the disease. Duchenne muscular dystrophy intertwines with bone loss and osteoporosis. Advances in dystrophinopathy diagnosis may allow the early detection of preclinical cases of muscular dystrophy and the disease prediction in infancy. Corticosteroids are a palliative therapy which treats the symptoms rather the cause of the disease rooted in the genomic DNA. Gene therapy holds the promise to correct *Dystrophin* gene alterations at their DNA roots, offering once in a lifetime therapy. While gene transfer of short versions of the *Dystrophin* gene could help all DMD patients, CRISPR gene editing, exon reframing, and exon skipping will help small subsets of DMD patients, which therefore will be considered personalized medicine. Furthermore, it is feasible that gene therapy for DMD and most of genetic diseases will be accessible within a decade. So, the authors predict a bright horizon for Duchenne muscular dystrophy gene therapy.
